# Targeted proteomics-derived biomarker profile develops a multi-protein classifier in liquid biopsies for early detection of esophageal squamous cell carcinoma from a population-based case-control study

**DOI:** 10.1186/s40364-021-00266-z

**Published:** 2021-02-17

**Authors:** Xiaorong Yang, Chen Suo, Tongchao Zhang, Xiaolin Yin, Jinyu Man, Ziyu Yuan, Jingru Yu, Li Jin, Xingdong Chen, Ming Lu, Weimin Ye

**Affiliations:** 1 Clinical Epidemiology Unit, Qilu Hospital of Shandong University, 107 Wenhuaxi Road, Jinan, 250012 Shandong China; 2 Clinical Research Center of Shandong University, Qilu Hospital of Shandong University, Jinan, China; 3 Department of Epidemiology and Health Statistics, School of Public Health, Fudan University, Shanghai, China; 4 Department of Epidemiology and Health Statistics, School of Public Health, Shandong University, Jinan, China; 5 Fudan University Taizhou Institute of Health Sciences, Taizhou, China; 6 Department of Medical Epidemiology and Biostatistics, Karolinska Institutet, Stockholm, Sweden; 7 State Key Laboratory of Genetic Engineering, Human Phenome Institute, and School of Life Sciences, Fudan University, Songhu Road 2005, Shanghai, 200438 China; 8 Department of Epidemiology and Health Statistics & Key Laboratory of Ministry of Education for Gastrointestinal Cancer, Fujian Medical University, Fuzhou, China

**Keywords:** Esophageal squamous cell carcinoma, Early detection, Diagnostic biomarkers, Affinity proteomics, Proximity extension assay, Screening

## Abstract

**Background:**

Early diagnosis of esophageal squamous cell carcinoma (ESCC) remains a challenge due to the lack of specific blood biomarkers. We aimed to develop a serum multi-protein signature for the early detection of ESCC.

**Methods:**

We selected 70 healthy controls, 30 precancerous patients, 60 stage I patients, 70 stage II patients and 70 stage III/IV ESCC patients from a completed ESCC case-control study in a high-risk area of China. Olink Multiplex Oncology II targeted proteomics panel was used to simultaneously detect the levels of 92 cancer-related proteins in serum using proximity extension assay.

**Results:**

We found that 10 upregulated and 13 downregulated protein biomarkers in serum could distinguish the early-stage ESCC from healthy controls, which were validated by the significant dose-response relationships with ESCC pathological progression. Applying least absolute shrinkage and selection operator (LASSO) regression and backward elimination algorithm, ANXA1 (annexin A1), hK8 (kallikrein-8), hK14 (kallikrein-14), VIM (vimentin), and RSPO3 (R-spondin-3) were kept in the final model to discriminate early ESCC cases from healthy controls with an area under curve (AUC) of 0.936 (95% confidence interval: 0.899 ~ 0.973). The average accuracy rates of the five-protein classifier were 0.861 and 0.825 in training and test data by five-fold cross-validation.

**Conclusions:**

Our study suggested that a combination of ANXA1, hK8, hK14, VIM and RSPO3 serum proteins could be considered as a potential tool for screening and early diagnosis of ESCC, especially with the establishment of a three-level hierarchical screening strategy for ESCC control.

**Supplementary Information:**

The online version contains supplementary material available at 10.1186/s40364-021-00266-z.

## Background

The International Agency for Research on Cancer estimated that there were 572,034 new esophageal cancer cases and 508,585 deaths from esophageal cancer worldwide in 2018, and the mortality-to-incidence ratios in most countries were more than 0.8 [1]. The 5-year overall survival rate of esophageal cancer ranges from 15 to 25% because most patients are diagnosed at an advanced stage with a dismal prognosis [2, 3]. Evidence demonstrates that the population-based screening programs for upper gastrointestinal cancers in East Asia could efficiently identify precancerous lesions and early cancers which leads to improved prognosis due to timely treatment [4, 5]. The government-sponsored endoscopic screening program is also conducted for asymptomatic adults in high incidence area for esophageal cancer in China, but the ongoing program introduced a large burden for public health: only a small part of residents could participate in the gastroscopy screening program and even among this small proportion of population long-term follow-up for high-risk subjects is becoming increasingly burdensome as regards endoscopic management [6]. Thus, a cost-effective and fast blood-based screening test (liquid biopsy) is an ideal solution for risk stratification in order to identify a truly high-risk population for endoscopy [7].

At present, various blood biomarkers including mutations and methylation status in cell-free DNA, cell-free RNA, noncoding RNAs, proteins, and so on, have been explored to fulfill the purpose of early detection of multiple cancer types via different detection platforms [8–11]. Esophageal squamous cell carcinoma (ESCC) is the dominant histological subtype of esophageal cancer (> 80% proportion), especially in Asia and Eastern African, [12] showing contrasting risk factors and molecular features with esophageal adenocarcinoma. To date, few effective biomarkers for screening early ESCC are established in clinical applications, because the area under curve (AUC) of most potential biomarkers is usually lower than 80% [8, 13].

In order to identify novel protein biomarkers, the development of proximity extension assays (PEA) has enabled simultaneous quantification of multiple targeted protein biomarkers for a bunch of samples in every experiment, thereby enabling quick screening of possible biomarkers. PEA innovatively combines the specificity of antibody-linked detection methods with the sensitivity of the polymerase chain reaction (PCR), permitting multiplex biomarker detection and quantification with reliable assay precision using only microliter quantities of sera [14]. Based on a prospectively designed population-based case-control study of upper gastrointestinal cancer in Taixing (with a population of about 1.13 million), a high-incidence area in China, this study applied the PEA technology to identify candidate serum protein biomarkers for early-stage ESCC.

## Methods

### Study design and participants

The research design of this large population-based case-control study has been delineated in our previous studies [15–18]. In brief, we attempted to recruit all newly diagnosed esophageal cancer cases from October 2010 to September 2013 in Taixing, and the inclusion criteria were limited to 40–85 year-old participants who had lived in Taixing at least 5 years. In the endoscopic units of the local four largest hospitals (covering almost 90% of local clinical diagnoses), the participants were invited to complete a questionnaire by trained interviewers and provided biological samples, if they were suspected of having an upper gastrointestinal tumor. Moreover, we further enrolled missed esophageal cancer patients in the endoscopy units by matching with the local Cancer Registry system. We finally recruited 1401 suspected esophageal cases from the hospitals’ endoscopy units and 280 reported esophageal cases via the local Cancer Registry system during 3 y. After reviewing the pathological sections and surgical pathology reports for those without pathological sections, 33 patients with precancerous lesions (high grade intraepithelial neoplasia, in-situ carcinoma, and high grade dysplasia) and 1418 ESCC patients were included in this study. Through evaluating the tumor stage of the 1418 ESCC patients via inpatient medical records based on the American Joint Commission on Cancer Staging Manual, 8th edition [19], we found additional 4 precancerous patients (reclassified from ESCC), 84 patients with stage I, 333 patients with stage II, 158 patients with stage III, 145 patients with stage IV and remaining 694 patients with unknown tumor stage. During the same period, we applied a frequency-matched method by sex and 5-year age groups to select control participants for the cases of upper gastrointestinal cancers. Finally, 1992 eligible controls participated in our study (participation rate: 70.4%).

The significant level of the hypothesis test was set as 0.001 for 92 proteins and the statistical power was set as 90%. As this study was dedicated to identifying high-efficiency serum protein biomarkers, it was estimated that the difference for significant biomarkers between patient group and control group should be at least 0.8 times of the standard deviation. The sample size of each group was calculated as 69, and we planned to select 70 subjects for each group.

For this study, we further limited suitable blood samples as those collected before clinical treatment and without moderate hemolysis. After excluding hemolytic samples and samples after treatment, the remaining 30 precancerous patients and 60 stage I patients were included. We then first randomly selected 70 patients from stage II ESCC patients, and randomly selected 70 advanced patients (stage III or stage IV) and 70 healthy controls by matching sex and 5-year age groups with stage II ESCC patients. If the sample size of patients in an age group was insufficient, it was supplemented from an adjacent age group.

**Fig. 1:**
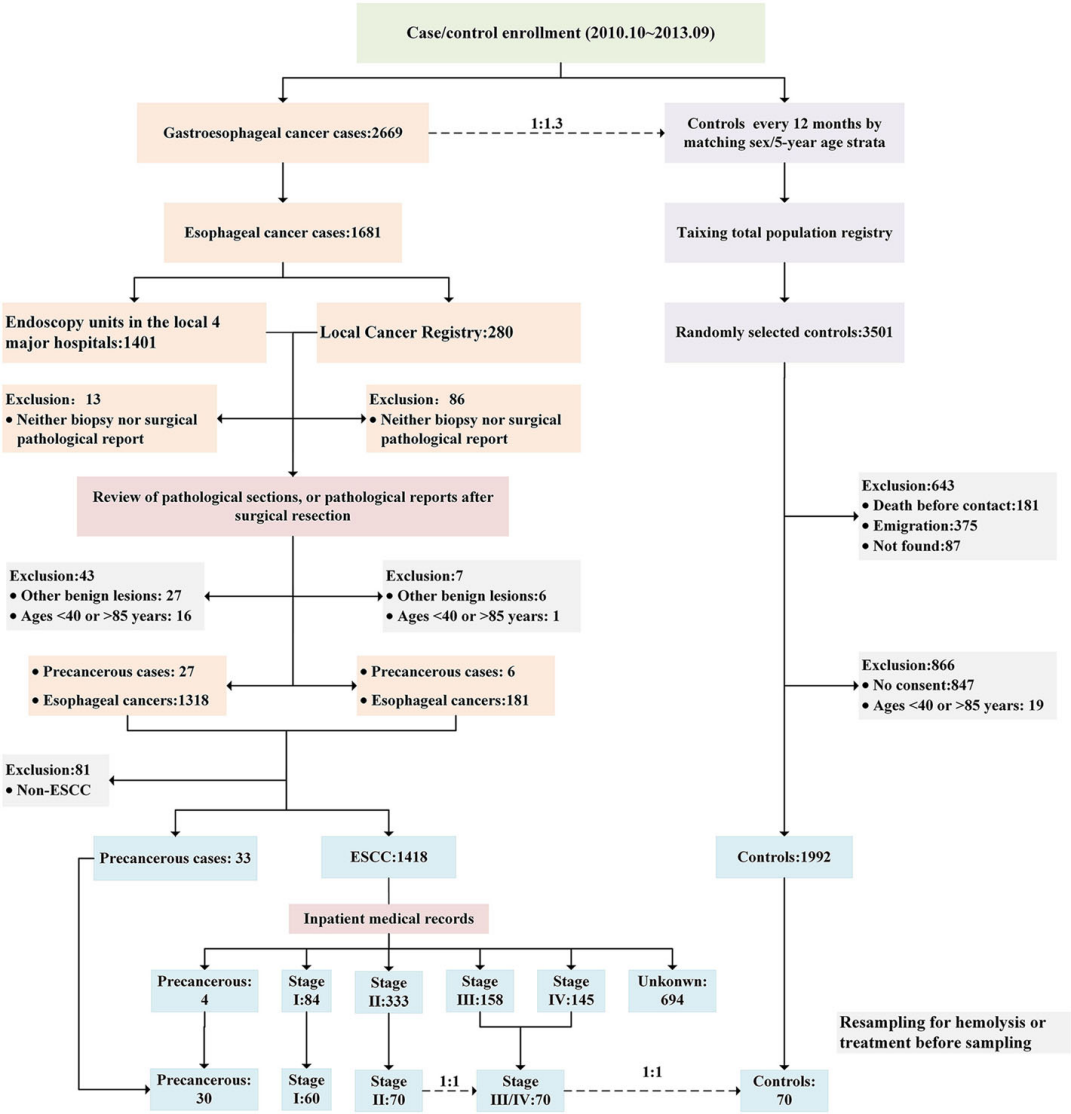
Selection diagram of participants enrolled in this study. ESCC, esophageal squamous cell carcinoma

We finally enrolled 30 precancerous patients, 60 stage I patients, 70 stage II patients, 70 stage III/IV patients and 70 healthy controls in the biomarker study (Fig. 1). The early-stage ESCC was defined as precancerous lesions and stage I cancer in our study because of mini-invasive treatment, better prognosis and small sample size, and early screening requirement in community-based practice [20]. Without external validation, we performed a dose-response relationship between serum protein levels and ESCC pathological progression to further illustrate the reliability of the identified biomarkers.

### Olink multiplex oncology II targeted proteomics panel

Serum proteins were analyzed using Olink Oncology II 96-well in which 92 oligonucleotide-labeled antibody probe pairs bind to their specific targeted proteins based on PEA technology [14, 21]. The precision, reproducibility and scalability of the PEA assay have been documented by the manufacturer (http://www.olink.com) and relevant articles [14, 21]. The protein names, gene names, and abbreviations for the 92 proteins of the Olink Oncology II panel are delineated in Table S1.

### Sample processing and detection

Blood samples have been stored in the − 80 °C refrigerator before shipment. The serum samples were shipped to Olink Proteomics AB (Uppsala, Sweden) using cold chains and the samples were randomly placed in four 96-well plates. On each plate, we included three “Inter-plate controls” for data normalization between plates and three “Negative controls” to establish background levels. Data generated from the plates were analyzed, including normalization and linearization, per manufacturer’s protocol. The protein levels were expressed as Normalized Protein eXpression (NPX) values, a relative quantification on a log scale, which are cycle threshold values normalized by the subtraction of values for the extension control. All assay characteristics including detection limits and measurements of assay performance and validation are available from the manufacturer’s website (http://www.olink.com/products/).

### Statistical methods

Chi-squared test or one-way ANOVA test were performed for testing the difference of the distributions of categorical or continuous variables in subgroups. An exploratory multivariate analysis (principal component analysis, PCA) was applied to test for potential clustering of study groups. The association between each protein NPX value and early ESCC was investigated using unconditional logistic regression, and the *P* value was adjusted by the Benjamini-Hochberg method for controlling the false discovery rate (FDR < 0.01). For the potential protein biomarkers, we further applied Spearman correlation to assess the dose-response relationship between protein levels and stages of ESCC, and the P value was also adjusted by the Benjamini-Hochberg method. For all preliminarily verified proteins, unsupervised clustering methods were applied to the data to identify clusters of proteins and visually evaluate their association with disease status. The Protein-Protein Interactions Network analysis of identified proteins was performed using the STRING database (https://string-db.org/). The online ConsensusPathDB-human interaction network database (http://cpdb.molgen.mpg.de/) was used for gene ontology (GO) enrichment analysis and pathway enrichment analysis of identified protein biomarkers. Three GO enrichment categories were checked, i.e. biological process, cellular component and molecular function.

For developing a multi-biomarker classifier to discriminate early ESCC cases from healthy controls, we used the least absolute shrinkage and selection operator (LASSO) regression to select optimal proteins. Moreover, we further used the backward elimination logistic regression model to build a more concise and efficient classification model. The specificity and sensitivity of the classifier were evaluated using the receiver operating characteristic (ROC) curve and the optimal cutoff points were selected using Youden’s index, which maximizes the sum of sensitivity and specificity. The AUC was applied to summarize the classification accuracy of diagnostic models and 95% confidence intervals (CI) were estimated by the non-parametric bootstrap. Five-fold cross-validation was used to estimate the validity of our multiple-protein model on the same data that was used to build the classifier. All statistical analyses and figure drawing were conducted using R (version 3.6.2).

## Results

### Patient overview and assay performance characteristics

The age and gender distribution were homogeneous among healthy controls and four groups of ESCC cases with different cancer stages in Table S2. The average intra-assay and inter-assay coefficient of variation (CV) based on quality control samples were 5 and 23% across the four plates, respectively.

### Prinicipal component analysis

**Fig. 2:**
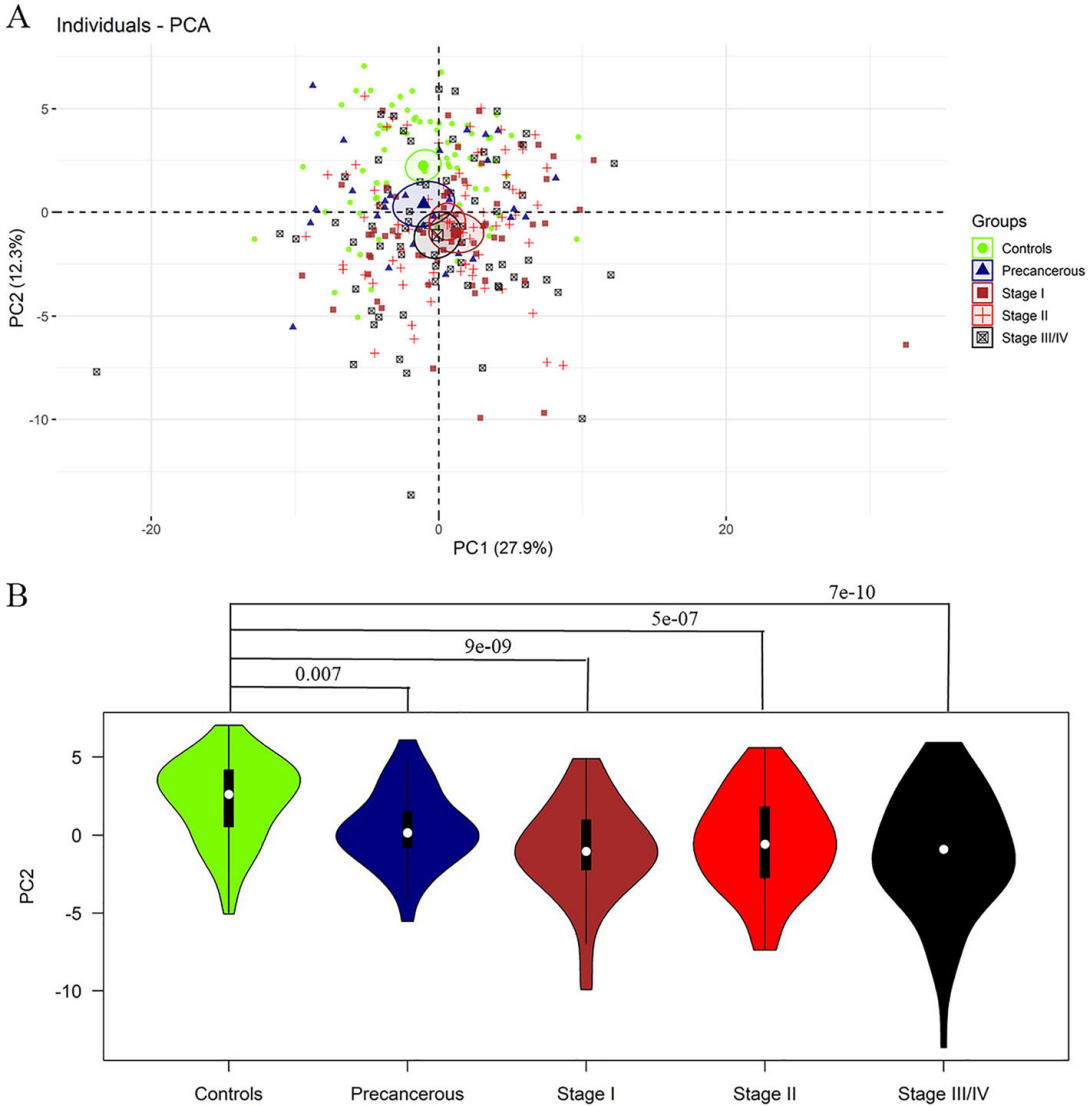
The distributions of proteins in different groups of participants. **a**, Distribution of dimension 1 (PC 1) and dimension 2 (PC 2) based on principal component analysis (PCA) of 92 proteins. **b**, ANOVA pairwise comparison with principal component 2 (PC2), compared with healthy controls

The results from principal component analysis of 70 healthy controls, 30 precancerous patients, 60 stage I patients, 70 stage II patients and 70 stage III/IV patients are shown in Fig. 2a. The numbers on the axes represent the variation captured by each principal component. The levels of 92 serum proteins were explained 40.2% by the first two principal components (PC1 27.9%, PC2 12.3%, respectively), and healthy controls were separated from ESCC patients with PC2. Thus, the PC2 distribution across various groups is illustrated in Fig. 2b, and ANOVA analysis found a significant difference among five groups (*P* = 2.5e-10) and pairwise comparison showed the healthy controls were significantly distinct from each ESCC group.

### Evaluation of diagnostic efficiency of each protein

**Fig. 3:**
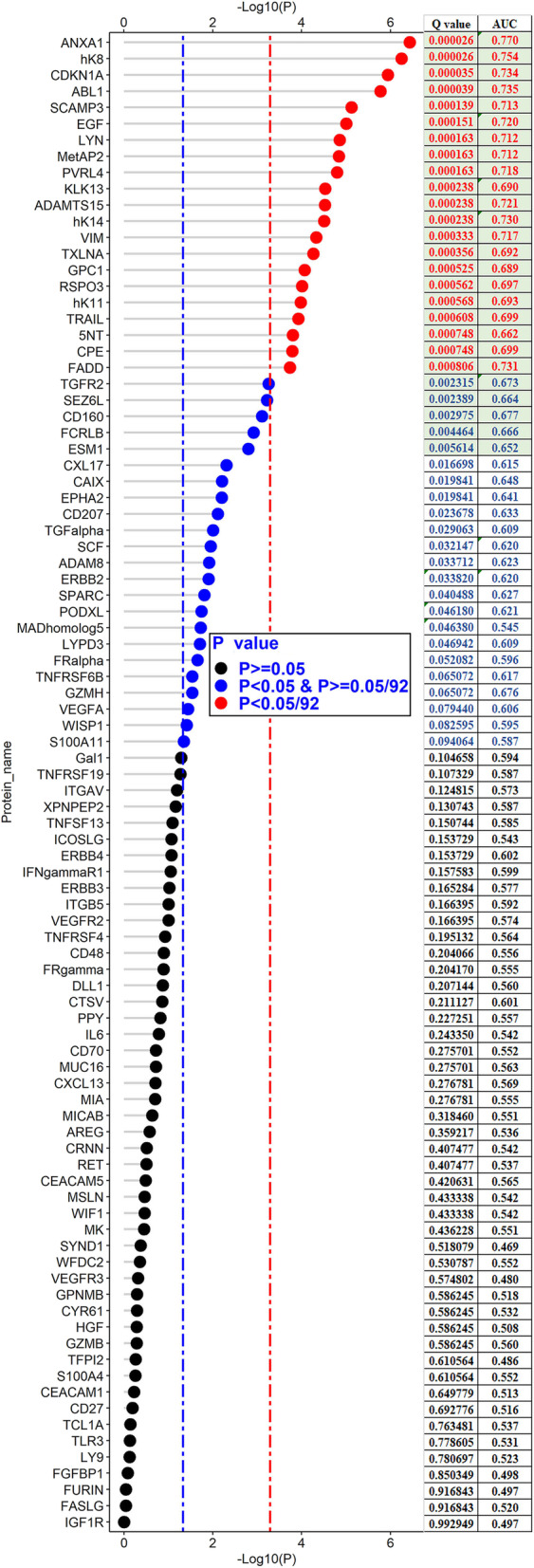
Dot plot of 92 proteins for distinguishing early esophageal squamous cell carcinoma from healthy controls, which presents *P* value, Q value adjusted by Benjamini-Hochberg method, and area of curve (AUC) for each protein

To identify potential protein biomarkers of early ESCC, the *P* value adjusted by FDR (Q value) and AUC of each protein for distinguishing healthy controls from early ESCC cases are shown in Fig. 3. According to the criterion of Q value less than 0.01, 26 potential proteins were discerned preliminarily, namely (sorting by Q value), ANXA1, hK8, CDKN1A, ABL1, SCAMP3, EGF, LYN, MetAP2, PVRL4, KLK13, ADAMTS15, hK14, VIM, TXLNA, GPC1, RSPO3, hK11, TRAIL, 5NT, CPE, FADD, TGFR2, SEZ6L, CD160, FCRLB, and ESM 1. The largest and smallest AUC of the 26 proteins were 0.770 for ANXA1 and 0.652 for ESM 1, respectively.

**Fig. 4:**
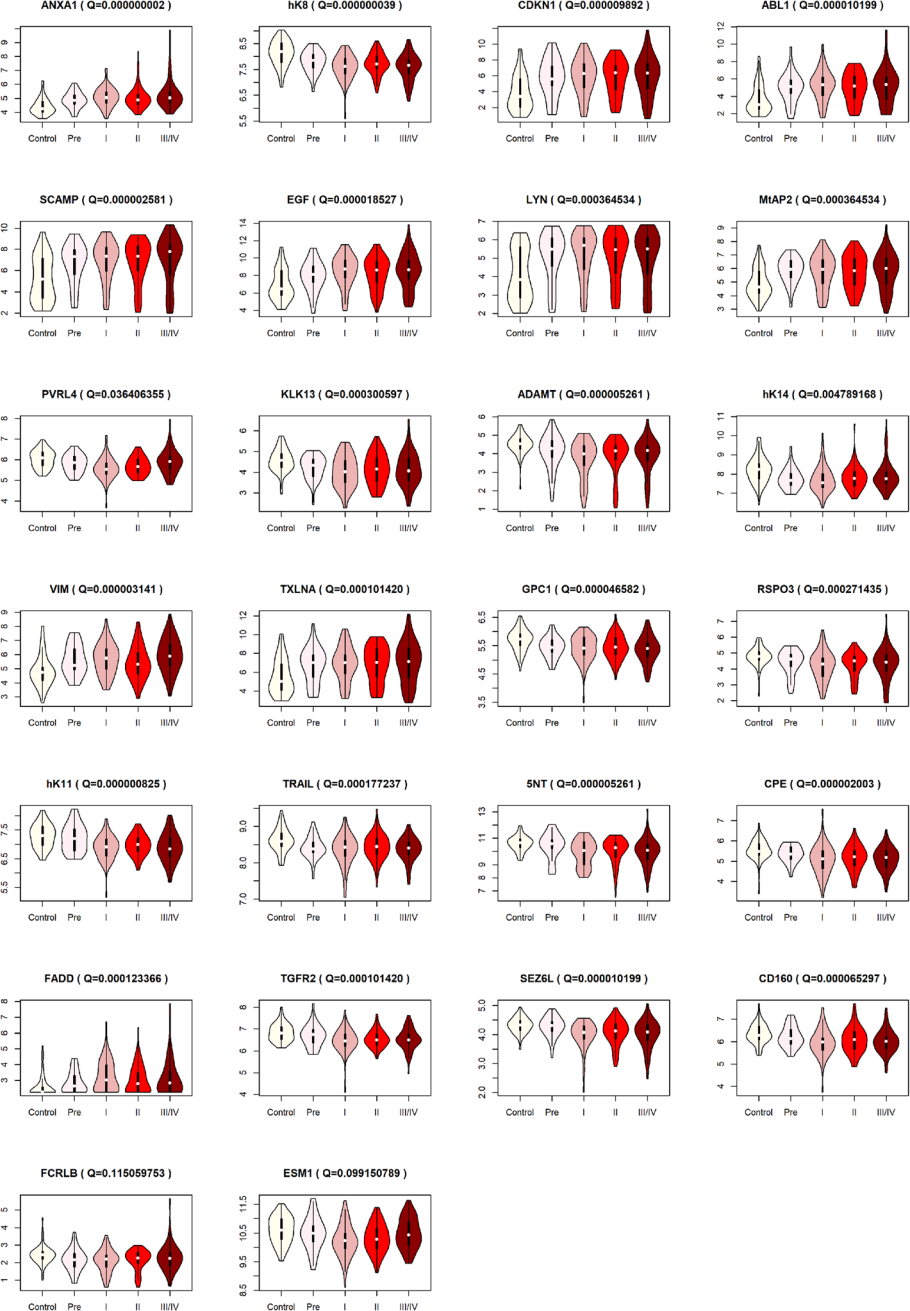
The distribution of 26 preliminarily identified proteins among five groups. Q value adjusted by Benjamini-Hochberg method stands for Spearman correlation between serum level of each protein and esophageal squamous cell carcinoma stages. Y-axis is NPX value of serum protein level of each value

For assessing the dose-response relationship between the levels of protein and the progression from healthy controls to advanced ESCC, the violin plots of different groups and *P* value of Spearman correlation adjusted by FDR (Q value) for these 26 proteins are displayed in Fig. 4. According to the criterion of Q values less than 0.01, remaining 10 upregulated (ANXA1, CDKN1A, ABL1, SCAMP3, EGF, LYN, MetAP2, VIM, TXLNA and FADD) and 13 downregulated (hK8, KLK13, ADAMTS15, hK14, GPC1, RSPO3, hK11, TRAIL, 5NT, CPE, TGFR2, SEZ6L and CD160) protein biomarkers in serum were authenticated as potential ESCC biomarkers.

### Protein interaction network and GO enrichment analyses

The protein interaction network analysis (Fig. S1) of 23 preliminarily authenticated proteins showed that 5NT, ABL1, ANXA1, CDKN1A, EGF, GPC1, hK11, hK14, hK8, KLK13, LYN, TGFR2, and VIM shared potential interactions. GO enrichment analysis further revealed that signaling receptor binding and catalytic activity, were the top ontologies for the ‘molecular function’ category, while extracellular space and extracellular organelle were the top ontologies for the ‘cellular component’ category, and negative regulation of response to stimulus and regulation of response to stimulus were the top enriched ontologies for the ‘biological process’ category (Table S3). Pathway enrichment analysis revealed that TP53 Network and Glypican 1 network were the top two enriched pathways (Table S4). An unsupervised hierarchical clustering analysis of 23 preliminarily authenticated proteins showed a significant distinction for early ESCC cases from healthy controls (Fig. S2).

### Creation of multi-protein diagnostic model

**Fig. 5:**
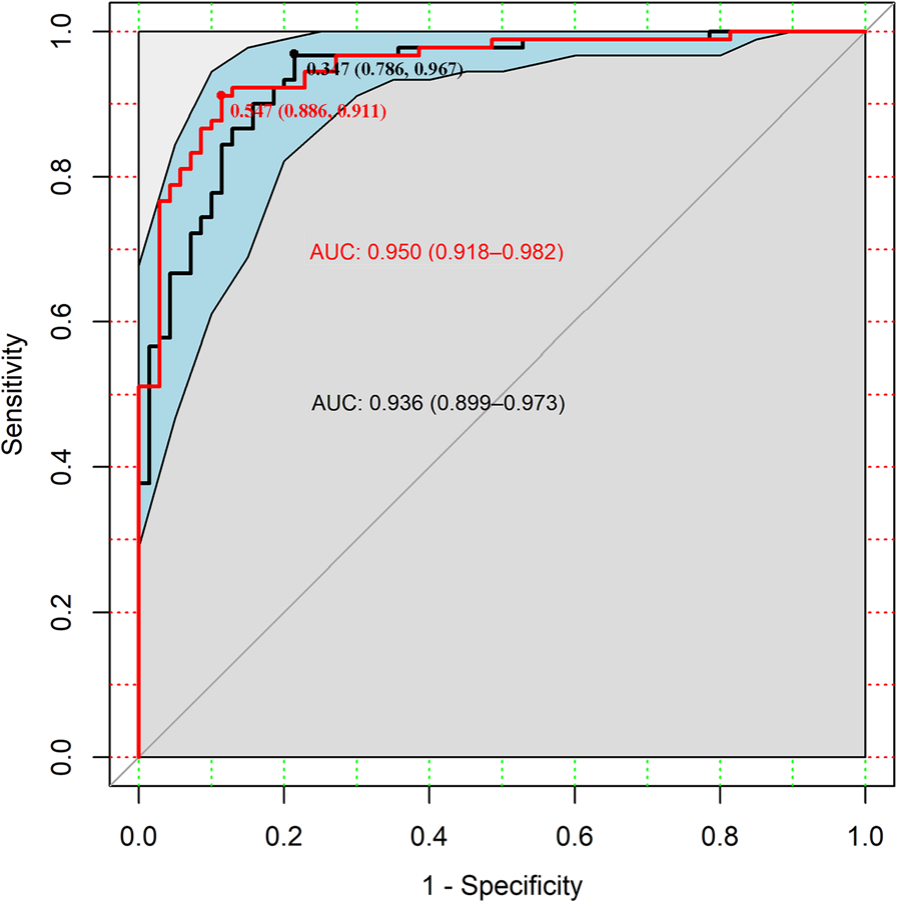
Receiver operating characteristic (ROC) curve for selected multiple protein classifier. The red line and area under the curve (AUC) value was fitted by 11 proteins model, namely, ANXA1, hK8, CDKN1A, MetAP2, hK14, VIM, GPC1, RSPO3, TRAIL, 5NT, SEZ6L. The black line with 95% confidence interval (CI) and AUC value was fitted by 5 compact proteins model, namely ANXA1, hK8, hK14, VIM and RSPO3. Diagnosis models were built by using logistic regression

Considering the complex relationship of these 23 proteins and clinical feasibility for using these biomarkers, LASSO regression was performed to select optimal proteins based on dimensionality reduction in order to develop a compact multi-protein classifier (Fig. S3). Remaining 11 proteins, namely, ANXA1, hK8, CDKN1A, MetAP2, hK14, VIM, GPC1, RSPO3, TRAIL, 5NT, and SEZ6L were used to construct a multiple logistic regression model with an AUC of 0.950 (95%CI:0.918 ~ 0.982, Fig. 5 red line). Because the model still had the problem of multicollinearity and redundant protein biomarkers, a backward elimination algorithm was further applied to construct a brief and efficient multi-protein model. Finally, ANXA1, hK8, hK14, VIM and RSPO3 were kept to discriminate early ESCC cases from healthy controls with an AUC of 0.936 (95%CI:0.899 ~ 0.973, Fig. 5 black line). The specificity and sensitivity of the classifier were 78.6 and 96.7% at optimal Youden’s index, and the classified accuracy was 0.888. Besides, the average accuracy rates of the five- protein model were 0.861 and 0.825 in training and test data by 5-fold cross-validation. For intuitive understanding, the results from logistic regression analysis for protein levels (quartiles) are shown in Table 1, and an easy-to-use predictive nomogram tool was created to evaluate the individual ESCC risk based on the five-protein panel (Fig. S4).

**Table 1:** The association of serum concentrations of five selected proteins with the risk of early ESCC

Protein	Controls	Cases	Crude OR95%CI	Adjusted OR(95%CI) ^**†**^
**ANXA1 as categorical variable**				
First quartile (low)	30	10	1.0 (referent)	1.0 (referent)
Second quartile	21	19	2.714 (1.071–7.209)	3.364 (0.722–17.31)
Third quartile	13	27	6.231 (2.351–16.513)	8.128 (1.403–57.13)
Fourth quartile (high)	6	34	17.00 (5.520–52.359)	31.76 (3.804–355.3)
*P* for trend			1.1e-07	0.0020
**hK8 as categorical variable**				
First quartile (low)	9	31	1.0 (referent)	1.0 (referent)
Second quartile	28	23	0.677 (0.242–1.840)	0.995 (0.197–4.927)
Third quartile	19	21	0.321 (0.118–0.826)	0.384 (0.067–2.013)
Fourth quartile (high)	30	10	0.097 (0.033–0.261)	0.034 (0.004–0.200)
*P* for trend			2.1e-06	0.0002
**hK14 as categorical variable**				
First quartile (low)	8	32	1.0 (referent)	1.0 (referent)
Second quartile	13	27	0.519 (0.181–1.418)	0.766 (0.158–3.576)
Third quartile	20	20	0.250 (0.089–0.655)	0.220 (0.041–1.025)
Fourth quartile (high)	29	11	0.095 (0.032–0.257)	0.054 (0.008–0.283)
*P* for trend			2.1e-06	0.0004
**VIM as categorical variable**				
First quartile (low)	26	14	1.0 (referent)	1.0 (referent)
Second quartile	25	15	1.114 (0.446–2.794)	6.818 (1.529–35.43)
Third quartile	11	29	4.896 (1.939–13.11)	14.87 (2.629–107.6)
Fourth quartile (high)	8	32	7.429 (2.802–21.50)	69.80 (7.297–1145.4)
*P* for trend			3.8e-06	0.0006
**RSPO3 as categorical variable**				
First quartile (low)	6	34	1.0 (referent)	1.0 (referent)
Second quartile	16	24	0.265 (0.084–0.744)	0.250 (0.042–1.312)
Third quartile	23	17	0.130 (0.041–0.362)	0.064 (0.008–0.359)
Fourth quartile (high)	25	15	0.106 (0.033–0.295)	0.017 (0.001–0.128)
*P* for trend			1.2e-05	0.0002

^**†**^The model contains the five listed serum proteins

The overall results did not change substantially, after conducting a sensitivity analysis by adjusting for age and sex in logistic regression models.

## Discussion

### Protein signatures comparing with existent studies

Proteomic studies have been conducted to explore potential biomarkers for ESCC diagnosis by using different biological samples, such as body fluids (plasma, serum, etc.), tumor tissues (fresh frozen tissues or formalin-fixed-paraffin-embedded tissues) and cells in vitro. In 2016, Harada et al. summarized 18 non-targeted proteomic studies with limited sample sizes for ESCC diagnosis based on mass spectrometry technology using serum, tissue and cell line samples, and identified several novel ESCC diagnostic markers, such as Apolipoprotein A-I, Tubulin beta chain filamin A alpha, HSP70, and so on [22]. Blood-based diagnostic studies have been extensively used as a cost-effective and fast screening tool for understanding diseases and medication treatment efficiency over the years, and organ-specific proteins in plasma could mirror organ dysfunction [23]. Development of a liquid biopsy method for early ESCC detection would significantly improve the efficiency of subsequent gastroscopy examination, especially for asymptomatic high-risk population.

Recently, a study identified 13 protein biomarkers in serum using the protein chip AAH-BLG-507 from RayBiotech for discriminating 10 early ESCC patients from 10 healthy controls in China [24]. Liao et al. reported that a combination of plasma FAPα plus traditional biomarker (CEA, CYFRA211, SCCA) using ELISA could significantly discriminate (AUC = 0.745) ESCC (*n* = 151, stage I: 29 + stage II: 59 + stage III/IV: 63) from non-malignancy controls (*n* = 230, healthy: 194 + benign:36) [25]. Huang et al. reported an AUC of 0.725 for serum IGFBP7 based on a study including 107 controls and 37 early ESCC patients [26]. Xu et al. reported the serum autoantibody panel (p53, MMP-7, HSP70, Prx VI and Bmi-1) could distinguish early-stage ESCC patients (*n* = 76) from normal controls (*n* = 134) with sensitivity of 45% and specificity of 96% in a validation cohort [13]. In our study, 23 proteins, namely, ANXA1, hK8, CDKN1A, ABL1, SCAMP3, EGF, LYN, MetAP2, KLK13, ADAMTS15, hK14, VIM, TXLNA, GPC1, RSPO3, hK11, TRAIL, X5NT, CPE, FADD, TGFR2, SEZ6L and CD160, showed potential diagnostic utility for distinguishing early ESCC from controls and their serum levels showed a significant dose-response relationship with ESCC stages. However, few overlapped proteins were found in the above-mentioned studies, which may be due to differences of candidate protein signatures, sample sizes, ESCC stages, biological nature of samples (plasma vs. serum) and detection methods (PEA vs. protein chip vs. ELISA) used in various studies.

This is the first study to estimate the efficiency of Olink Oncology II panel for the early diagnosis of ESCC. Although this panel was not designed specifically for identifying ESCC patients, the majority of the proteins on the Oncology II panel are secreted proteins that show abnormal expression in the tissues or sera of multiple types of cancer [21, 27, 28]. Especially, several proteins, such as, ANXA1, CEACAM5 (aka CEA), VIM, ALB1 and IL6, have been reported to be potential biomarkers in the diagnosis of ESCC, [24, 25, 29–32] however, most proteins on the Oncology II panel have not yet been examined for their expression in ESCC blood samples.

### Model performance

In order to avoid overfitting and consider the clinical feasibility for early diagnosis of ESCC, a concise multi-protein classifier containing ANXA1, hK8, hK14, VIM and RSPO3 was created. The AUC of the five-protein classifier for differentiating early ESCC from controls was 0.936 (95%CI:0.899 ~ 0.973). The specificity and sensitivity were 78.6 and 96.7% at optimal Youden’s index, and the classification accuracy was 0.888. We used five-fold cross-validation to estimate the average accuracy rate of the five-protein classifier, and the corresponding figure was 0.861 and 0.825 in the discovery set and validation set, respectively. Overall, the differentiation efficiency of our multi-protein classifier was relatively superior to other studies [13, 25, 26, 33].

### Biological functions

In our study, 92 tumor-related candidate proteins were detected in serum from various stage ESCC patients and healthy controls to predict cancer status, and 23 proteins were preliminarily identified as potential diagnostic protein biomarkers for ESCC. Functional enriched pathway analyses of these 23 proteins showed that they were involved in signaling receptor binding, extracellular space, regulation of response to stimulus and TP53 network implicated in development of ESCC. Thus, their compositions in serum could mirror the pro-tumorigenic ESCC microenvironment and can be used to monitor the progression of ESCC.

In our final diagnostic classifier for early stage ESCC, ANXA1, hK8, hK14, VIM and RSPO3 were selected. The serum levels of ANXA1 and VIM were over-expressed in ESCC patients, on the contrary, the serum levels of hK8, hK14 and RSPO3 were decreased.

ANXA1 (annexin A1), known as an endogenous anti-inflammatory protein, has now been recognized to be closely related to tumor cell proliferation, invasion, differentiation, apoptosis, metastasis and chemotherapy sensitivity via modulation of various cancer-associated pathways [34, 35]. Moreover, ANXA1 shows contrasting expression profiles in various cancer types: over-expressed in lung cancer, colorectal cancer, and pancreatic cancer, and so on, by the contrary, lack of expression in cervical cancer, prostate cancer, nasopharyngeal carcinoma, etc. [34, 36] We found a high level of ANXA1 in serum of ESCC patients, which is consistent with the finding of a previous study showing upregulated levels of ANXA1 in ESCC tissues versus matching normal tissues [30]. However, most previous studies reported that ANXA1 expression was significantly downregulated in cell lines and tissues from ESCC patients compared with adjacent normal tissues [29, 32, 37–39]. Further studies are needed to examine the correlation of tumor ANXA1 expression with serum level.

VIM (vimentin), one of class-III intermediated filament proteins, is involved with cytoskeletal integrity, cell adhesion and cell migration via epithelial-mesenchymal transition, [40, 41] and upregulated VIM levels in tissues have been reported as a potential diagnostic and prognostic marker of multiple types of cancers, such as prostate cancer, breast cancer, malignant melanoma and lung cancer [42]. The over-expressed VIM was reported in ESCC tissues compared with adjacent normal tissues, [30] which was somewhat consistent with the results of our study. The biological expression of vimentin is regulated by the transcription factors Twist, Zeb1, Snail, and Slug, which are induced by TGF-β signal transduction [43].

Dysregulation of kallikrein-related peptidases (KLKs) is related to differential expression signatures in various types of cancers, [44, 45] but little is known about its role in ESCC development. Four proteins from kallikrein-related peptidase family, namely, hK8(kallikrein-8), hK11(kallikrein-11), KLK13(kallikrein-13) and hK14(kallikrein-14), were detected by Olink Oncology II panel, and we found all of them had low levels in serum in ESCC patients regardless tumor stage, compared with healthy controls. KLKs, the largest secreted serine protease family, are involved in cancer cell growth, migration, invasion, and chemo-resistance by activation of PARs, the release of active growth factors, modulation of the proteolytic network, and activation of androgen receptor signaling [45, 46].

RSPO3 (R-spondin-3), an activator of the canonical Wnt signaling pathway and PI3K/AKT pathway as a key regulator of angiogenesis and epithelial-mesenchymal transition, has shown low expression in colorectal cancer, squamous cell carcinoma of the lung and prostate cancer, but upregulated expression in bladder cancer, ovarian cancer and lung adenocarcinoma [47–50]. Our study showed that RSPO3 level in serum was inversely associated with ESCC progression.

### Limitations and future perspectives

The results of our models should be interpreted with caution. First, the study was conducted in an ESCC high-risk area of China, which might weaken the generalization of our five-protein prediction classifier to other relatively normal-risk areas. Second, although we found the overall good dose-response relationship between the serum levels of identified biomarkers and ESCC stages, the trends of certain proteins were not perfect, which recommends that external, independent studies are needed to validate and generalize our findings. Moreover, the identified protein biomarkers for ESCC were generally universal biomarkers for multiple types of tumors. Further work is needed to determine the specificity of our five-protein classifier for ESCC diagnosis versus other cancer types. Considering a three-level hierarchical screening strategy, i.e. “environment exposure + blood biopsy + esophagogastroduodenoscopy”, to be established in ESCC high-incidence area, our serum multi-protein classifier with high sensitivity and specificity would have a promising application value in high-risk population. The identified ESCC biomarkers are also involved in ESCC progression, which highlights their possible application also as prognostic biomarkers.

In summary, we identified and established a multi-protein classifier for discriminating early ESCC patients from healthy controls, which might contribute to improving the three-level hierarchical screening strategy for decreasing the ESCC burden in high-incidence areas. However, the results need to be further validated in prospective cohort studies.

## Supplementary Information

**Additional file 1: Table S1.** 92 proteins from the Olink multiplex Oncology II panel. **Table S2.** The general information of selected participants, controls and cases based on different cancer stages. **Figure S1.** The protein interaction of 23 preliminarily authenticated proteins. Each node represents a protein, and the gene name is marked at the top right of the node. **Table S3.** Gene ontology enrichment analysis of the identified 23 proteins that were differentially expressed between early ESCC and controls, covering three categories, i.e. molecular function, cellular component, and biological process. Top 5 gene ontologies in each enrichment category were selected. Data were obtained from the online ConsensusPathDB- human interaction network database http://cpdb.molgen.mpg.de/. **Table S4.** Pathway enrichment analysis of the identified 23 proteins that were differentially expressed between early ESCC and controls. Top 7 enriched pathway were selected. Data were obtained from the online ConsensusPathDB- human interaction network database http://cpdb.molgen.mpg.de/. **Figure S2.** An unsupervised hierarchical clustering analysis of 23 preliminarily authenticated proteins for discriminating early esophageal squamous cell carcinoma (ESCC) from healthy controls. **Figure S3.** The selection feature of least absolute shrinkage and selection operator (LASSO) via tenfold cross-validation based on area under the ROC curve (AUC). Selection of the tuning parameter (λ) in the LASSO model was via tenfold cross-validation based on minimum standard error. The y-axis indicates AUC. The lower x-axis indicates the log(λ). Numbers along the upper x-axis represent the average number of predictors. Red dots indicate average AUC values for each model with a given λ, and vertical bars through the red dots show the upper and lower values of AUC. The vertical black lines define the optimal values of λ, where the model provides its best fit to the data. **Figure S4.** A nomogram to predict individual ESCC risk based on the identified five-protein panel.

## Data Availability

All data that support the findings of this study are available from the corresponding authors upon a reasonable request.

## Abbreviations

ESCC: Esophageal squamous cell carcinoma
LASSO: Least absolute shrinkage and selection operator
ANXA1: Annexin A1
hK8: Kallikrein-8
hK14: Kallikrein-14
VIM: Vimentin
RSPO3: R-spondin-3
AUC: Area under curve
PEA: Proximity extension assays
PCR: Polymerase chain reaction
NPX: Normalized Protein eXpression
PCA: Principal component analysis
FDR: False discovery rate
GO: Gene ontology
ROC: Receiver operating characteristic
CI: Confidence intervals
CV: Coefficient of variation
PC: Principal components
hK11: Kallikrein-11
KLK13: Kallikrein-13
