# Supplementary material for: Targeted proteomics-derived biomarker profile develops a multi-protein classifier in liquid biopsies for early detection of esophageal squamous cell carcinoma from a population-based case-control study
Source: Biomark Res. 2021 Feb 17;9:12. doi: 10.1186/s40364-021-00266-z (PMC7890600; doi:10.1186/s40364-021-00266-z)

**Targeted proteomics-derived biomarker profile develops a multi-protein classifier in liquid biopsies for early detection of esophageal squamous cell carcinoma from a population-based case-control study**

**Running title:** S**erum protein biomarkers for early detection of ESCC**

**Xiaorong Yang, Chen Suo, Tongchao Zhang, Xiaolin Yin, Jinyu Man, Ziyu Yuan, Jingru Yu, Li Jin, Xingdong Chen*, Ming Lu*, Weimin Ye**

***Correspondence and requests for materials should be addressed to:**

Ming Lu at Clinical Epidemiology Unit, Qilu Hospital of Shandong University, 107 Wenhuaxi Road, Jinan, Shandong 250012, China. Tel: +86-531-82169051; Fax: +86-531-86927544; Email: [lvming@sdu.edu.cn](mailto:lvming@sdu.edu.cn)

Xingdong Chen, State Key Laboratory of Genetic Engineering, Human Phenome Institute, and School of Life Sciences, Fudan University, Songhu Road 2005, Shanghai 200438, China. Tel.: +86-21-51630602, Fax: +86-21-51630607. E-mail: [xingdongchen@fudan.edu.cn](mailto:xingdongchen@fudan.edu.cn)

**Supplementary tables: 4; Supplementary figures:4**

**List of supporting information**

**Table S1.** 92 proteins from the Olink multiplex Oncology II panel.

**Table S2.** The general information of selected participants, controls and cases based on different cancer stages.

**Figure S1.** The protein interaction of 23 preliminarily authenticated proteins. Each node represents a protein, and the gene name is marked at the top right of the node.

**Table S3.** Gene ontology enrichment analysis of the identified 23 proteins that were differentially expressed between early ESCC and controls, covering three categories, i.e. molecular function, cellular component, and biological process. Top 5 gene ontologies in each enrichment category were selected. Data were obtained from the online ConsensusPathDB- human interaction network database <http://cpdb.molgen.mpg.de/>.

**Table S4.** Pathway enrichment analysis of the identified 23 proteins that were differentially expressed between early ESCC and controls. Top 7 enriched pathway were selected. Data were obtained from the online ConsensusPathDB- human interaction network database <http://cpdb.molgen.mpg.de/>.

**Figure S2.** An unsupervised hierarchical clustering analysis of 23 preliminarily authenticated proteins for discriminating early esophageal squamous cell carcinoma (ESCC) from healthy controls.

**Figure S3.** The selection feature of least absolute shrinkage and selection operator (LASSO) via tenfold cross-validation based on area under the ROC curve (AUC). Selection of the tuning parameter (λ) in the LASSO model was via tenfold cross-validation based on minimum standard error. The y-axis indicates AUC. The lower x-axis indicates the log(λ). Numbers along the upper x-axis represent the average number of predictors. Red dots indicate average AUC values for each model with a given λ, and vertical bars through the red dots show the upper and lower values of AUC. The vertical black lines define the optimal values of λ, where the model provides its best fit to the data.

**Figure S4.** A nomogram to predict individual ESCC risk based on the identified five-protein panel.

**Table S1.** 92 proteins from the Olink multiplex Oncology II panel.

| Item | Protein name | Uniprot | Gene name | Description | Protein name we used |
| --- | --- | --- | --- | --- | --- |
| 1 | DLL1 | O00548 | DLL1 | Delta like canonical Notch ligand 1 | DLL1 |
| 2 | PODXL | O00592 | PODXL | Podocalyxin like | PODXL |
| 3 | CYR61 | O00622 | CYR61 | Cysteine rich angiogenic inducer 61 | CYR61 |
| 4 | SCAMP3 | O14828 | SCAMP3 | Secretory carrier-associated membrane protein 3 | SCAMP3 |
| 5 | TLR3 | O15455 | TLR3 | Toll-like receptor 3 | TLR3 |
| 6 | XPNPEP2 | O43895 | XPNPEP2 | Xaa-Pro aminopeptidase 2 | XPNPEP2 |
| 7 | CXCL13 | O43927 | CXCL13 | C-X-C motif chemokine ligand 13 | CXCL13 |
| 8 | hK8 | O60259 | KLK8 | Kallikrein related peptidase 8 | hK8 |
| 9 | CTSV | O60911 | CTSV | Cathepsin L2 | CTSV |
| 10 | ICOSLG | O75144 | ICOSLG | Inducible T cell costimulator ligand; ICOS ligand | ICOSLG |
| 11 | TNFSF13 | O75888 | TNFSF13 | Tumor necrosis factor ligand superfamily member 13 | TNFSF13 |
| 12 | LYPD3 | O95274 | LYPD3 | Ly6/PLAUR domain-containing protein 3 | LYPD3 |
| 13 | WISP-1 | O95388 | WISP1 | WNT1-inducible-signaling pathway protein 1 | WISP1 |
| 14 | TNFRSF6B | O95407 | TNFRSF6B | Tumor necrosis factor receptor superfamily member 6B | TNFRSF6B |
| 15 | CD160 | O95971 | CD160 | CD160 antigen | CD160 |
| 16 | ABL1 | P00519 | ABL1 | ABL proto-oncogene 1, non-receptor tyrosine kinase | ABL1 |
| 17 | EGF | P01133 | EGF | Pro-epidermal growth factor | EGF |
| 18 | TGF-alpha | P01135 | TGFA | Transforming growth factor alpha | TGFalpha |
| 19 | PPY | P01298 | PPY | Pancreatic prohormone | PPY |
| 20 | ANXA1 | P04083 | ANXA1 | Annexin A1 | ANXA1 |
| 21 | ErbB2/HER2 | P04626 | ERBB2 | Receptor tyrosine-protein kinase erbB-2 | ERBB2 |
| 22 | IL6 | P05231 | IL6 | Interleukin-6 | IL6 |
| 23 | CEA | P06731 | CEACAM5 | Carcinoembryonic antigen-related cell adhesion molecule 5 | CEACAM5 |
| 24 | ITGAV | P06756 | ITGAV | Integrin subunit alpha V | ITGAV |
| 25 | LYN | P07948 | LYN | LYN proto-oncogene, Src family tyrosine kinase | LYN |
| 26 | RET | P07949 | RET | Proto-oncogene tyrosine-protein kinase receptor Ret | RET |
| 27 | IGF1R | P08069 | IGF1R | Insulin-like growth factor 1 receptor | IGF1R |
| 28 | VIM | P08670 | VIM | Vimentin | VIM |
| 29 | CD48 | P09326 | CD48 | CD48 antigen | CD48 |
| 30 | Gal-1 | P09382 | LGALS1 | Galectin-1 | Gal1 |
| 31 | SPARC | P09486 | SPARC | Secreted protein acidic and cysteine rich | SPARC |
| 32 | FUR | P09958 | FURIN | Furin, paired basic amino acid cleaving enzyme | FURIN |
| 33 | GZMB | P10144 | GZMB | Granzyme B | GZMB |
| 34 | CEACAM1 | P13688 | CEACAM1 | Carcinoembryonic antigen-related cell adhesion molecule 1 | CEACAM1 |
| 35 | HGF | P14210 | HGF | Hepatocyte growth factor | HGF |
| 36 | IFN-gamma-R1 | P15260 | IFNGR1 | Interferon gamma receptor 1 | IFNgammaR1 |
| 37 | FR-alpha | P15328 | FOLR1 | Folate receptor alpha | FRalpha |
| 38 | AREG | P15514 | AREG | Amphiregulin | AREG |
| 39 | VEGF-A | P15692 | VEGFA | Vascular endothelial growth factor A | VEGFA |
| 40 | CPE | P16870 | CPE | Carboxypeptidase E | CPE |
| 41 | ITGB5 | P18084 | ITGB5 | Integrin subunit beta 5 | ITGB5 |
| 42 | SYND1 | P18827 | SDC1 | Syndecan-1 | SYND1 |
| 43 | GZMH | P20718 | GZMH | Granzyme H | GZMH |
| 44 | SCF | P21583 | KITLG | Stem cell factor | SCF |
| 45 | 5'-NT | P21589 | NT5E | 5'-nucleotidase ecto | 5NT |
| 46 | MK | P21741 | MDK | Midkine | MK |
| 47 | ErbB3/HER3 | P21860 | ERBB3 | Receptor tyrosine-protein kinase erbB-3 | ERBB3 |
| 48 | S100A4 | P26447 | S100A4 | S100 calcium binding protein A4 | S100A4 |
| 49 | CD27 | P26842 | CD27 | CD27 antigen | CD27 |
| 50 | EPHA2 | P29317 | EPHA2 | Ephrin type-A receptor 2 | EPHA2 |
| 51 | S100A11 | P31949 | S100A11 | S100 calcium binding protein A11 | S100A11 |
| 52 | CD70 | P32970 | CD70 | CD70 antigen | CD70 |
| 53 | GPC1 | P35052 | GPC1 | Glypican-1 | GPC1 |
| 54 | VEGFR-3 | P35916 | FLT4 | Vascular endothelial growth factor receptor 3 | VEGFR3 |
| 55 | VEGFR-2 | P35968 | KDR | Vascular endothelial growth factor receptor 2 | VEGFR2 |
| 56 | TGFR-2 | P37173 | TGFBR2 | Transforming growth factor beta receptor 2 | TGFR2 |
| 57 | CDKN1A | P38936 | CDKN1A | Cyclin-dependent kinase inhibitor 1 | CDKN1A |
| 58 | TXLNA | P40222 | TXLNA | Alpha-taxilin | TXLNA |
| 59 | FR-gamma | P41439 | FOLR3 | Folate receptor gamma | FR_gamma |
| 60 | TNFRSF4 | P43489 | TNFRSF4 | Tumor necrosis factor receptor superfamily member 4 | TNFRSF4 |
| 61 | FasL | P48023 | FASLG | Fas antigen ligand | FASLG |
| 62 | TFPI-2 | P48307 | TFPI2 | Tissue factor pathway inhibitor 2 | TFPI2 |
| 63 | MetAP 2 | P50579 | METAP2 | Methionine aminopeptidase 2 | MetAP2 |
| 64 | TRAIL | P50591 | TNFSF10 | TNF-related apoptosis-inducing ligand | TRAIL |
| 65 | TCL1A | P56279 | TCL1A | T-cell leukemia / lymphoma protein 1A | TCL1A |
| 66 | ADAM8 | P78325 | ADAM8 | Disintegrin and metalloproteinase domain-containing protein 8 | ADAM8 |
| 67 | FADD | Q13158 | FADD | FAS-associated death domain protein | FADD |
| 68 | MSLN | Q13421 | MSLN | Mesothelin | MSLN |
| 69 | WFDC2 | Q14508 | WFDC2 | WAP four-disulfide core domain protein 2 | WFDC2 |
| 70 | FGF-BP1 | Q14512 | FGFBP1 | Fibroblast growth factor-binding protein 1 | FGFBP1 |
| 71 | GPNMB | Q14956 | GPNMB | Transmembrane glycoprotein NMB | GPNMB |
| 72 | ErbB4/HER4 | Q15303 | ERBB4 | Receptor tyrosine-protein kinase erbB-4 | ERBB4 |
| 73 | MIA | Q16674 | MIA | Melanoma-derived growth regulatory protein | MIA |
| 74 | CAIX | Q16790 | CA9 | Carbonic anhydrase IX | CAIX |
| 75 | MIC-A/B | Q29983 Q29980 | MICA MICB | MHC class I polypeptide-related sequence A; MHC class I polypeptide-related sequence B | MICAB^†^ |
| 76 | FCRLB | Q6BAA4 | FCRLB | Fc receptor-like B | FCRLB |
| 77 | CXL17 | Q6UXB2 | CXCL17 | VEGF-co regulated chemokine 1 | CXL17 |
| 78 | ADAM-TS 15 | Q8TE58 | ADAMTS15 | A disintegrin and metalloproteinase with thrombospondin motifs 15 | ADAMTS15 |
| 79 | MUC-16 | Q8WXI7 | MUC16 | Mucin 16, cell surface associated | MUC16 |
| 80 | PVRL4 | Q96NY8 | NECTIN4 | Nectin cell adhesion molecule 4 | PVRL4 |
| 81 | MAD homolog 5 | Q99717 | SMAD5 | Mothers against decapentaplegic homolog 5 | MADhomolog_5 |
| 82 | RSPO3 | Q9BXY4 | RSPO3 | R-spondin-3 | RSPO3 |
| 83 | SEZ6L | Q9BYH1 | SEZ6L | Seizure related 6 homolog like | SEZ6L |
| 84 | LY9 | Q9HBG7 | LY9 | T-lymphocyte surface antigen Ly-9 | LY9 |
| 85 | ESM-1 | Q9NQ30 | ESM1 | Endothelial cell-specific molecule 1 | ESM1 |
| 86 | TNFRSF19 | Q9NS68 | TNFRSF19 | Tumor necrosis factor receptor superfamily member 19 | TNFRSF19 |
| 87 | hK14 | Q9P0G3 | KLK14 | Kallikrein related peptidase 14 | hK14 |
| 88 | CRNN | Q9UBG3 | CRNN | Cornulin | CRNN |
| 89 | hK11 | Q9UBX7 | KLK11 | Kallikrein related peptidase 11 | hK11 |
| 90 | CD207 | Q9UJ71 | CD207 | C-type lectin domain family 4 member K | CD207 |
| 91 | KLK13 | Q9UKR3 | KLK13 | Kallikrein related peptidase 13 | KLK13 |
| 92 | WIF-1 | Q9Y5W5 | WIF1 | Wnt inhibitory factor 1 | WIF1 |
| ^†^Both MIC_A and MIC_B were measured using one pair of antibodies because they are closely related and share about 85% amino acid similarity. | | | | | |

| **Table S2.** The general information of selected participants, controls and cases based on different cancer stages. | | | | | | |
| --- | --- | --- | --- | --- | --- | --- |
|  | **Controls**  **(N=70)**  **N (%)** | **Precancerous**  **(N=30)**  **N (%)** | **Stage I**  **(N=60**  **N (%)** | **Stage II**  **(N=70)**  **N (%)** | **Stage III/IV**  **(N=70)**  **N (%)** | ***P* value^†^** |
| **Age (mean±SD, years)** | 64.6±8.1 | 68.5±8.4 | 63.8±8.1 | 64.4±7.5 | 64.6±8.1 | 0.095 |
| **Gender** |  |  |  |  |  |  |
| Male | 46(65.7) | 18(60.0) | 42(70.0) | 46(65.7) | 46(65.7) | 0.922 |
| Female | 24(34.3) | 12(40.0) | 18(30.0) | 24(34.3) | 24(34.3) |  |
| Abbreviations: SD, standard deviation; N, number.  ^†^ *P* values were derived using analysis of variance test for continuous variables, and Chi-squared test or Fisher’s exact test for categorical variables. | | | | | | |

**
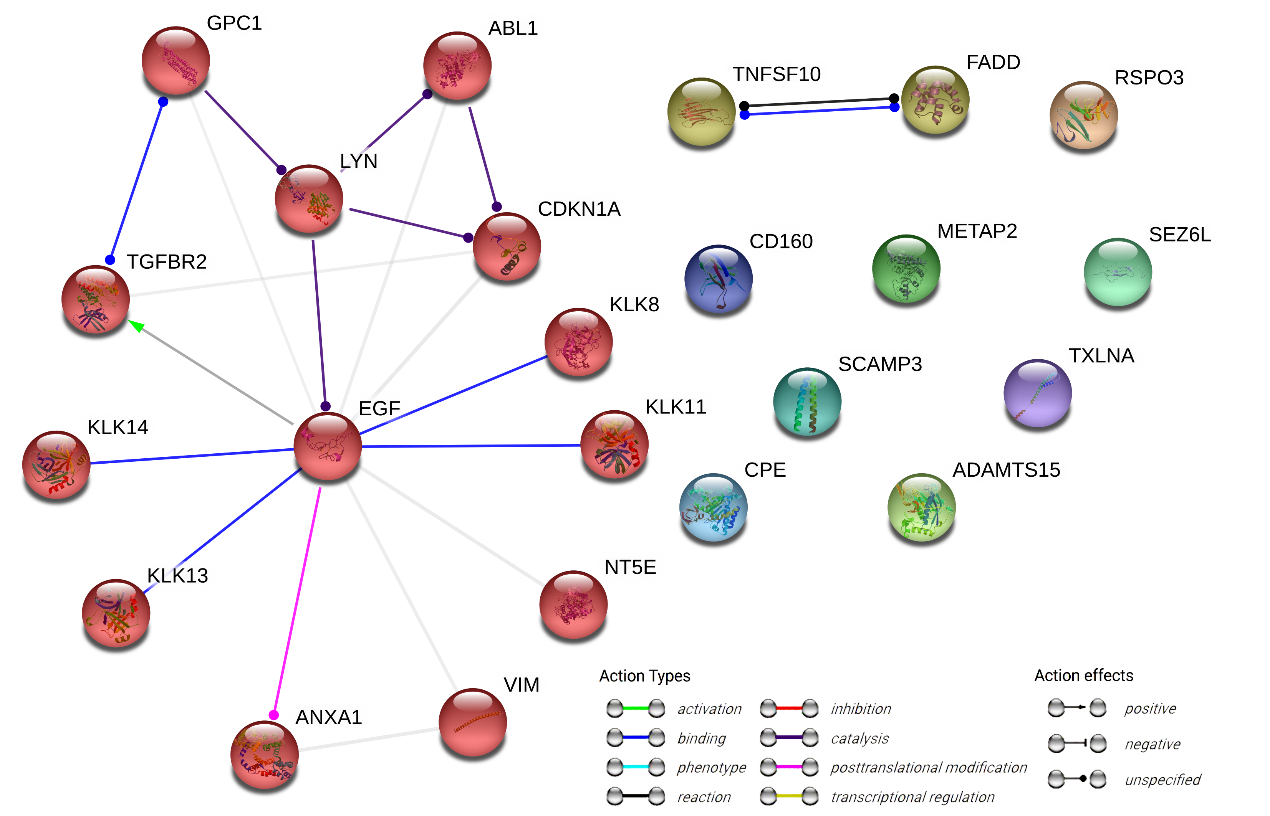
**

**Figure S1.** The protein interaction of 23 preliminarily authenticated proteins. Each node represents a protein, and the gene name is marked at the top right of the node.

**Table S3**. Gene ontology enrichment analysis of the identified 23 proteins that were differentially expressed between early ESCC and controls, covering three categories, i.e. molecular function, cellular component, and biological process. Top 5 gene ontologies in each enrichment category were selected. Data were obtained from the online ConsensusPathDB- human interaction network database <http://cpdb.molgen.mpg.de/>.

| **Category** | **FDR** | **Gene symbol** |
| --- | --- | --- |
| ***Molecular function*** | | |
| Signaling receptor binding | 0.0000276 | *ABL1; ANXA1; CD160; CPE; EGF; FADD; LYN; RSPO3; TGFBR2; TNFSF10; TXLNA;* |
| Catalytic activity, acting on a protein | 0.0000838 | *ABL1; ADAMTS15; CDKN1A; CPE; EGF; KLK8; KLK11; KLK13; KLK14; METAP2; LYN; TGFBR2* |
| Peptidase activity | 0.0002203 | *ADAMTS15,CPE, KLK8, KLK11, KLK13, KLK14, METAP2* |
| Peptidase activity, acting on L-amino acid peptides | 0.0003052 | *ADAMTS15,CPE, KLK8, KLK11, KLK13, KLK14, METAP2* |
| Serine-type endopeptidase activity | 0.0020384 | *KLK8; KLK13; KLK11; KLK14* |
|  | | |
| ***Cellular component*** | | |
| Extracellular space | 0.000016 | *ADAMTS15; ANXA1; CPE; EGF; GPC1; KLK8; KLK11; KLK13; KLK14; LYN; NT5E; RSPO3; SCAMP3; TNFSF10; VIM;* |
| Extracellular organelle | 0.001616 | *ANXA1; CPE; EGF; GPC1; KLK11; LYN; NT5E; SCAMP3; TNFSF10; VIM;* |
| Extracellular exosome | 0.001660 | *ANXA1; CPE; EGF; GPC1; KLK11; LYN; NT5E; SCAMP3; TNFSF10; VIM;* |
| Cytoplasmic vesicle | 0.001660 | *ANXA1; CPE; EGF; GPC1; KLK8; KLK11; KLK13; KLK14; SCAMP3; VIM;* |
| Extracellular vesicle | 0.001780 | *ANXA1; CPE; EGF; GPC1; KLK11; LYN; NT5E; SCAMP3; TNFSF10; VIM;* |
|  | | |
| ***Biological process*** | | |
| Negative regulation of response to stimulus | 0.0000091 | *ABL1; ANXA1; CDKN1A; EGF; FADD; GPC1; KLK8; KLK14; LYN; NT5E; TGFBR2; TNFSF10;* |
| Regulation of response to stimulus | 0.0000233 | *ABL1; ANXA1; CD160; CDKN1A; EGF; FADD; GPC1; KLK8; KLK14; LYN; METAP2; NT5E; RSPO3; SEZ6L; TGFBR2; TNFSF10; TXLNA;* |
| Animal organ development | 0.0000368 | *ABL1; ANXA1; CDKN1A; CPE; EGF; FADD; GPC1; KLK13; KLK14; LYN; RSPO3; SEZ6L; TGFBR2; TNFSF10; VIM;* |
| System development | 0.0000368 | *ABL1; ANXA1; CD160; CDKN1A; CPE; EGF; FADD; GPC1; KLK8; KLK13; KLK14; LYN; RSPO3; SEZ6L; TGFBR2; TNFSF10; VIM;* |
| Cell surface receptor signaling pathway | 0.0000368 | *ABL1; ANXA1; CD160; CDKN1A; CPE; EGF; FADD; GPC1; LYN; RSPO3; TGFBR2; TNFSF10; TXLNA; VIM;* |
| FDR, false discovery rate, adjusted by Benjamini-Hochberg method | | |

**Table S4**. Pathway enrichment analysis of the identified 23 proteins that were differentially expressed between early ESCC and controls. Top 7 enriched pathway were selected. Data were obtained from the online ConsensusPathDB- human interaction network database <http://cpdb.molgen.mpg.de/>.

| **Enriched pathway** | **FDR** | **Gene symbol** | **Pathway source** |
| --- | --- | --- | --- |
| TP53 network | 0.000619508 | *ABL1; CDKN1A; TNFSF10* | Wikipathways |
| Glypican 1 network | 0.000702063 | *TGFBR2; LYN; GPC1* | PID |
| Cyclin D associated events in G1 | 0.001503924 | *ABL1; LYN; CDKN1A* | Reactome |
| G1 Phase | 0.001503924 | *ABL1; LYN; CDKN1A* | Reactome |
| FoxO signaling pathway | 0.001503924 | *TGFBR2; CDKN1A; TNFSF10; EGF* | KEGG |
| EGFR1 | 0.001503924 | *ANXA1; SCAMP3; VIM; ABL1; LYN; EGF* | NetPath |
| TRAIL signaling | 0.001503924 | *TNFSF10; FADD* | Reactome |
| FDR, false discovery rate, adjusted by Benjamini-Hochberg method | | | |


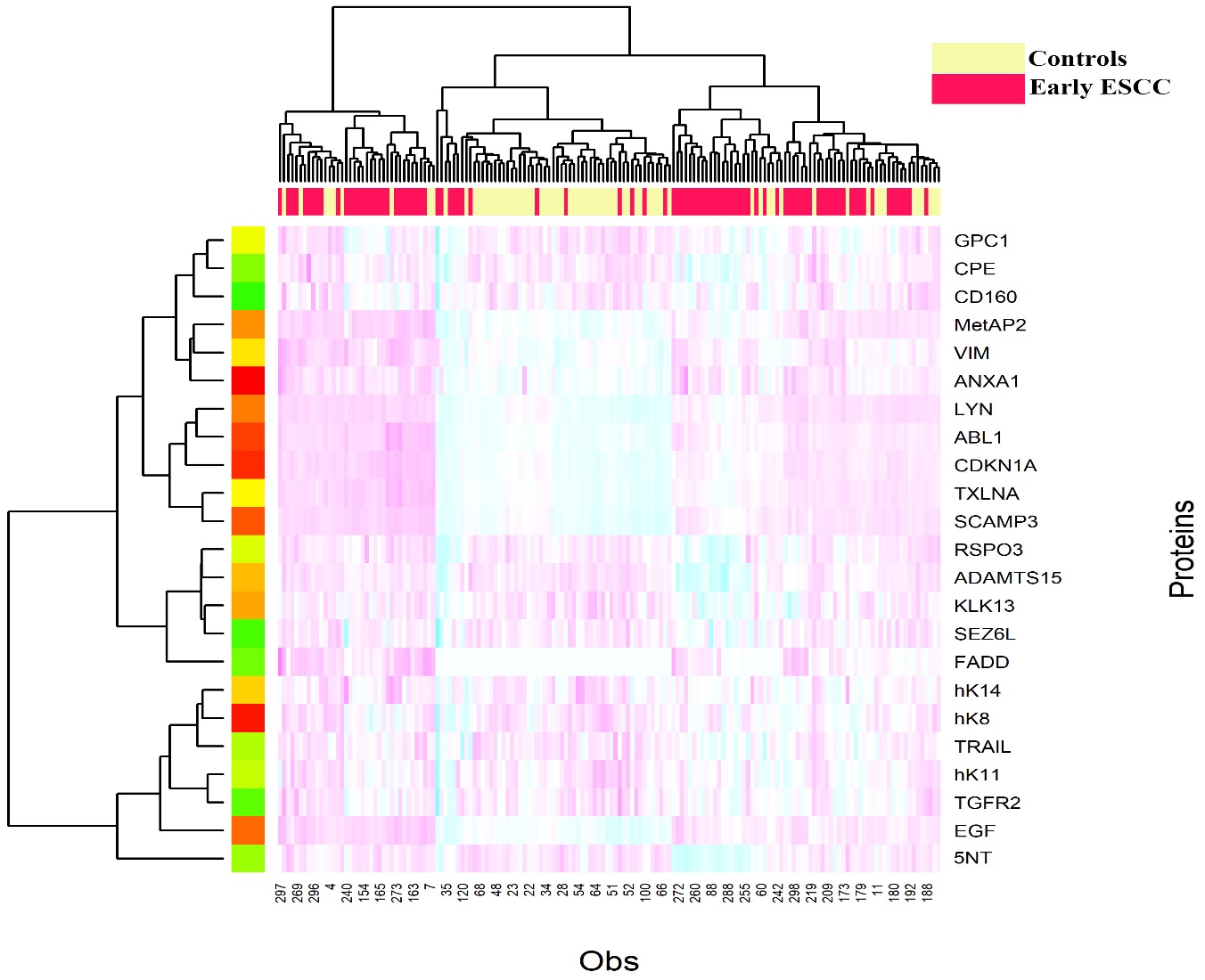


**Figure S2.** An unsupervised hierarchical clustering analysis of 23 preliminarily authenticated proteins for discriminating early esophageal squamous cell carcinoma (ESCC) from healthy controls.


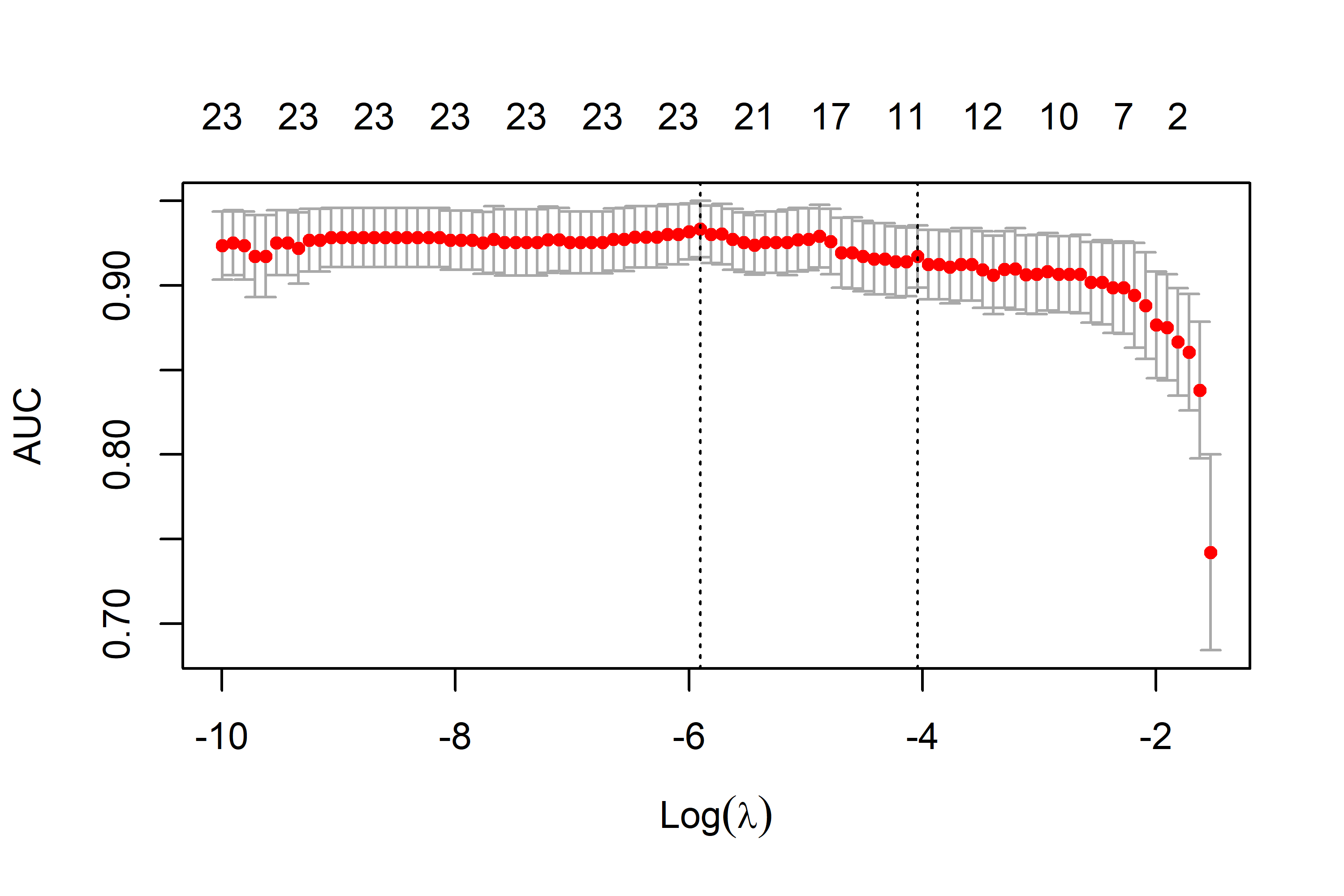


**Figure S3.** The selection feature of least absolute shrinkage and selection operator (LASSO) via tenfold cross-validation based on area under the ROC curve (AUC). Selection of the tuning parameter (λ) in the LASSO model was via tenfold cross-validation based on minimum standard error. The y-axis indicates AUC. The lower x-axis indicates the log(λ). Numbers along the upper x-axis represent the average number of predictors. Red dots indicate average AUC values for each model with a given λ, and vertical bars through the red dots show the upper and lower values of AUC. The vertical black lines define the optimal values of λ, where the model provides its best fit to the data.

**Figure S4.** A nomogram to predict individual ESCC risk based on the identified five-protein panel.


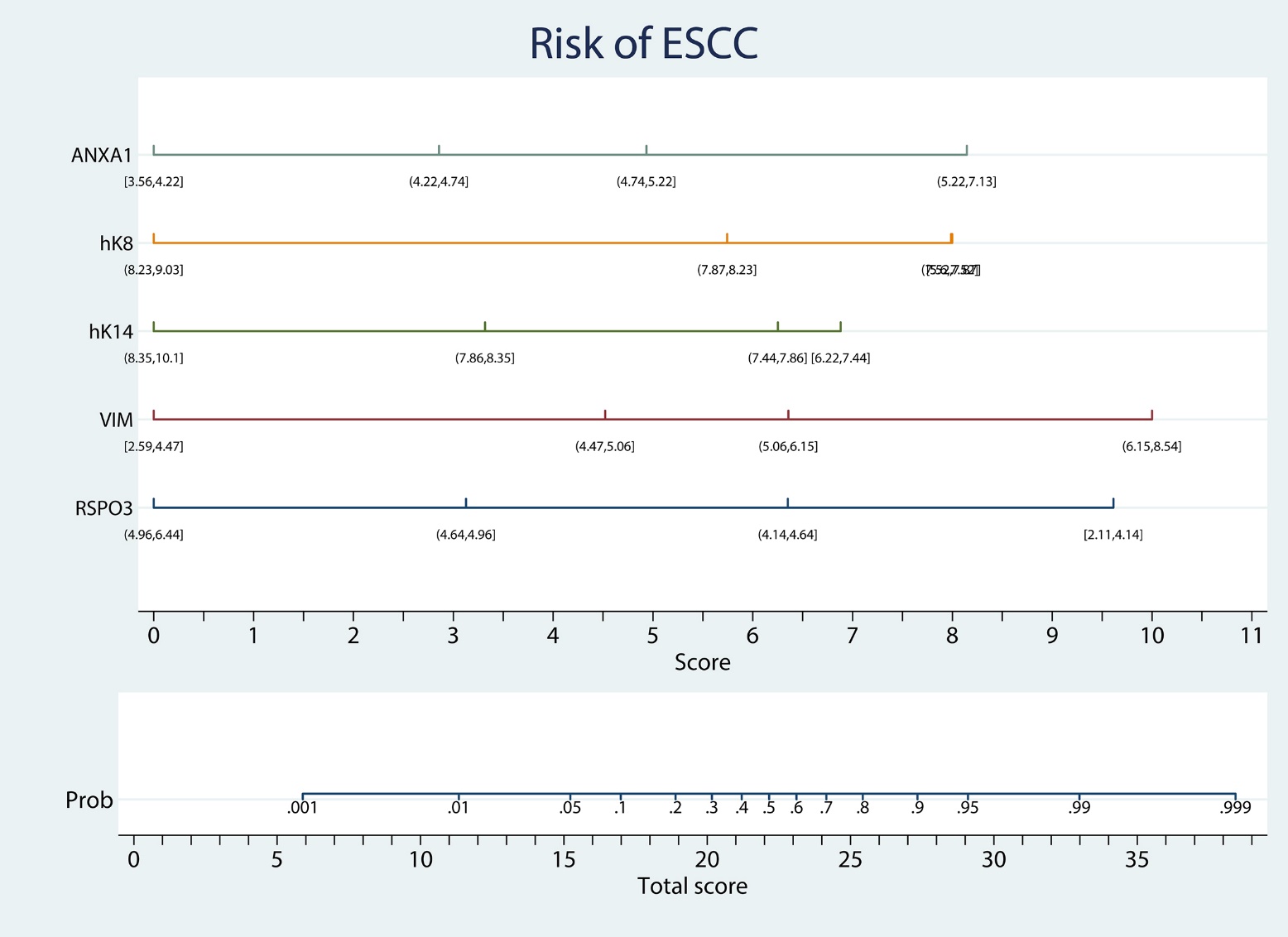

Supplement: Supplementary file 1 — Additional file 1: Table S1. 92 proteins from the Olink multiplex Oncology II panel. Table S2. The general information of selected participants, controls and cases based on different cancer stages. Figure S1. The protein interaction of 23 preliminarily authenticated proteins. Each node represents a protein, and the gene name is marked at the top right of the node. Table S3. Gene ontology enrichment analysis of the identified 23 proteins that were differentially expressed between early ESCC and controls, covering three categories, i.e. molecular function, cellular component, and biological process. Top 5 gene ontologies in each enrichment category were selected. Data were obtained from the online ConsensusPathDB- human interaction network database http://cpdb.molgen.mpg.de/. Table S4. Pathway enrichment analysis of the identified 23 proteins that were differentially expressed between early ESCC and controls. Top 7 enriched pathway were selected. Data were obtained from the online ConsensusPathDB- human interaction network database http://cpdb.molgen.mpg.de/. Figure S2. An unsupervised hierarchical clustering analysis of 23 preliminarily authenticated proteins for discriminating early esophageal squamous cell carcinoma (ESCC) from healthy controls. Figure S3. The selection feature of least absolute shrinkage and selection operator (LASSO) via tenfold cross-validation based on area under the ROC curve (AUC). Selection of the tuning parameter (λ) in the LASSO model was via tenfold cross-validation based on minimum standard error. The y-axis indicates AUC. The lower x-axis indicates the log(λ). Numbers along the upper x-axis represent the average number of predictors. Red dots indicate average AUC values for each model with a given λ, and vertical bars through the red dots show the upper and lower values of AUC. The vertical black lines define the optimal values of λ, where the model provides its best fit to the data. Figure S4. A nomogram to predict individual ESCC risk [file 40364_2021_266_MOESM1_ESM.docx]
